# Editorial: Psychopathological and behavioral trajectories in transitional-age youth: innovative approaches and paradigms

**DOI:** 10.3389/fnbeh.2026.1958484

**Published:** 2026-09-07

**Authors:** Michele Poletti, Gianluca Castelnuovo, Lorenzo Pelizza

**Affiliations:** 1 Department of Mental Health and Pathological Addictions, Istituto di Ricovero, Cura a Carattere Scientifico (IRCCS) Local Health Authority of Reggio Emilia, Reggio Emilia, Italy; 2 Department of Psychology, Catholic University of the Sacred Heart, Milano, Italy; 3 Department of Biomedical and Neuromotor Sciences, Alma Mater Studiorum University of Bologna, Bologna, Italy

**Keywords:** adolescence, development, psychopathology, psychosis, trajectory, transitional psychiatry, youth & adolescence

The transition from adolescence to adulthood represents a critical period, marked by significant psychological, behavioral, and neurobiological changes.

It is during this crucial period that individuals are most vulnerable to the emergence of severe mental disorders. Despite considerable progress in preclinical research aimed at understanding the neurobiological and genetic underpinnings of these conditions, there remains a significant gap in the continuity of care between child/adolescent and adult mental health services. This discontinuity often leads to service disengagement and under-treatment, exacerbating the mental health burden during this vulnerable period. This Research Topic seeks to explore the psychopathological and behavioral trajectories from childhood/adolescent neurodevelopmental syndromes to adult mental disorders and to describe innovative early intervention strategies tailored to the unmet needs of transitional-age youth. In this regard, the contributions collected in this Research Topic can be organized around *five* partially overlapping thematic clusters, each highlighting a different facet of psychopathological and behavioral trajectories in transitional-age youth.

The first cluster concerns the *epidemiology* and multi-layered determinants of youth mental health. Pang et al. provide the broadest population-level frame, documenting the increasing global burden of mental disorders among individuals aged 10–24 years between 1990 and 2021, with a marked rise in anxiety and depressive disorders, especially among females and during the COVID-19 period. Jiang et al. move from the global to the psychosocial level, showing in a large sample of university students how childhood adversities, family conflict, social support, and sleep disturbances are interconnected in shaping anxiety and depression. Larson et al. further refine this ecological perspective by distinguishing different dimensions of adversity, suggesting that threat exposure and resource scarcity do not operate as generic risks but interact in specific ways, with caregiver achievement-related scarcity moderating internalizing and externalizing outcomes. Kang et al., through their study of severe acute poisoning in adolescents, add a public health dimension to this cluster, emphasizing the frequent association between self-poisoning, female sex, psychiatric history, and suicidal intent. Together, these studies show that youth psychopathology is not reducible to individual vulnerability but emerges from cumulative and interacting biological, familial, social, and contextual pressures.

A second cluster focuses on internalizing symptoms, *transdiagnostic* mechanisms, and cognitive-affective processes. Jiang et al. again contribute to this area by identifying sleep disorders as proximal correlates of anxiety and depression. Li et al. use network analysis and Bayesian directed acyclic graphs to examine the links between mental health problems and attention deficit, showing that anxiety, interpersonal sensitivity, paranoia, hostility, and emotional instability are closely connected with inattention, hyperactivity/impulsivity, and oppositional behaviors. Chiappini et al. broaden the clinical understanding of depression in young people by reviewing novel depressive subtypes and emphasizing phenomena such as boredom, shame, fatigue, alexithymia, emotional dysregulation, social disconnection, substance use, gender dysphoria, and environmental stress. Zheng et al. examine the relationship between adolescent depression and moral thinking, suggesting that depressive symptoms may affect both deontological and utilitarian tendencies, partly through rumination, paranoia, and hostility. Mousavi et al. bring this cluster into the therapeutic field, showing that both internet-based and face-to-face transdiagnostic treatments may reduce anxiety and depression in adolescents, with sustained benefits in the online condition. Overall, these studies suggest that anxiety and depression in youth should be understood not simply as discrete syndromes, but as nodes within broader networks involving attention, social cognition, rumination, moral reasoning, affect regulation, and daily functioning.

A third and particularly substantial cluster addresses psychosis, schizophrenia-spectrum vulnerability, salience, and *developmental* nosology. Kong et al. examine diagnostic trajectories from non-severe to severe mental illness in hospitalized adolescents, showing that diagnostic instability and transition toward severe mental illness are especially relevant over medium-term follow-up. Wallman et al. explore digital early detection of clinical high risk for psychosis, highlighting both the promise and the methodological difficulty of online screening, particularly regarding data validity. Domenicano et al. focus on first-episode psychosis, showing sex-related differences in clinical presentation, substance use, functional recovery, and NEET status over follow-up. Baccaredda Boy et al. conceptualize early psychosis as a process of multi-domain de-structuring involving personality, reality testing and developmental organization, particularly in adolescents. Jeličić et al. contribute a phenomenological comparison between autism and schizophrenia-spectrum conditions, emphasizing both overlap and qualitative differences, especially in relation to “ipseity” disturbances and primary intersubjectivity. Ricci et al. revisit dissociation, automatism, and temporality in substance-induced and primary psychoses, offering phenomenological tools to differentiate exogenous and endogenous psychotic experiences in the context of new psychoactive drugs. Poletti et al. critically discuss the “HiTOP psychosis super-spectrum,” questioning whether dimensional models are sufficient without a genuinely developmental and psychopathological account. Finally, Merola et al. investigate creativity, aberrant salience, obsessive beliefs, and openness in artists, suggesting a nuanced connection between creative behavior and psychosis-proneness. This cluster is central to the Research Topic because it treats psychosis not as a fixed diagnostic endpoint, but as a developmental, phenomenological, neurocognitive, social, and service-related trajectory.

A fourth cluster concerns body, action, and *behavioral expression* as privileged languages of adolescent distress and transformation. The work by Kang et al. on acute poisoning illustrates how suicidal and self-harming behavior may become embodied crisis communication. Demaria et al. offer a developmental reflection on aggressiveness, presenting it as an ambivalent phenomenon that can support self-assertion and individuation, but may also become destructive when disconnected from empathy, reality testing, and social norms. Panarello et al. examine pharmacological treatment trajectories in hospitalized patients with eating disorders, linking psychopharmacological strategies with changes in body mass index (BMI) and psychopathological burden. The study conducted by Han and Choi examines ultimate frisbee as a group-based intervention, showing that sport participation may increase self-esteem and reduce social anxiety through group belonging. Taken together, these articles remind us that in transitional-age youth, psychopathology is often expressed through the body, action, risk, control, aggression, and eating behavior, while these same domains may also become therapeutic entry points.

A fifth cluster cuts across the previous ones and concerns *innovation* in detection, treatment, and service models. Wallman et al. address the opportunities and pitfalls of online screening for psychosis risk. Mousavi et al. demonstrate the feasibility of internet-based transdiagnostic treatment for adolescent anxiety and depression. Domenicano et al. show the importance of early intervention services in tracking functional outcomes after first-episode psychosis. Panarello et al. provide real-world evidence on treatment patterns in eating disorders, while Han and Choi illustrate the potential of community and group-based interventions. These contributions share a pragmatic concern: how to translate developmental psychopathology into accessible, timely, scalable, and youth-sensitive interventions.

Taken as a whole, the Research Topic suggests several interesting conclusions. First, transitional age should not be treated as a mere administrative boundary between child/adolescent and adult services, but as a developmental window with specific risks, opportunities, and clinical forms. Second, diagnostic categories remain necessary, but they are insufficient unless integrated with trajectories, mechanisms, context, and phenomenological quality. Third, youth mental health requires hybrid models combining epidemiology, psychopathology, digital innovation, early intervention, and social ecology. Finally, the heterogeneity of these contributions is not a limitation but a strength: it reflects the complexity of contemporary youth psychopathology and points toward a plural, developmentally informed, and clinically actionable paradigm.

